# Effect of downregulated citrate synthase on oxidative phosphorylation signaling pathway in HEI-OC1 cells

**DOI:** 10.1186/s12953-022-00196-0

**Published:** 2022-09-07

**Authors:** Xiaowen Xu, Yue Liu, Jun Luan, Rongrong Liu, Yan Wang, Yingying Liu, Ang Xu, Bingxin Zhou, Fengchan Han, Wenjing Shang

**Affiliations:** 1 Key Laboratory for Genetic Hearing Disorders in Shandong, Binzhou Medical University, 346 Guanhai Road, Yantai, 264003 Shandong People’s Republic of China; 2 Department of Otolaryngology, Yantai Affiliated Hospital of Binzhou Medical University, 717 Jinbu Road of Muping District, Yantai, 264100 Shandong People’s Republic of China; 3 Department of anesthesiology, Yantai Affiliated Hospital of Binzhou Medical University, 717 Jinbu Road of Muping District, Yantai, 264100 Shandong People’s Republic of China; 4 Department of Biochemistry and Molecular Biology, Binzhou Medical University, 346 Guanhai Road, Yantai, 264003 Shandong People’s Republic of China

**Keywords:** Citrate synthase, iTRAQ proteomics, Oxidative phosphorylation

## Abstract

**Background:**

Citrate Synthase (*Cs*) gene mutation (locus *ahL4*) has been found to play an important role in progressive hearing loss of A/J mice. HEI-OC1 cells have been widely used as an in vitro system to study cellular and molecular mechanisms related to hearing lose. We previously reported the increased apoptosis and the accumulation of reactive oxygen species in shRNA*Cs*-1429 cells, a *Cs* low-expressed cell model from HEI-OCI. The details of the mechanism of ROS production and apoptosis mediated by the abnormal expression of *Cs* needed to research furtherly.

**Methods:**

iTRAQ proteomics was utilized to detect the differentially expressed proteins (DEPs) caused by low expression of *Cs*. The GO and KEGG pathways analysis were performed for annotation of the differentially expressed proteins. Protein–protein interaction network was constructed by STRING online database. Immunoblotting was utilized to confirm the protein levels of the the differentially expressed proteins.

**Results:**

The differentially expressed proteins were significantly enriched in various signaling pathways mainly related to mitochondrial dysfunction diseases including Parkinson’s disease, Alzheimer’s disease, Huntington’s disease, et al. Most noteworthy, the oxidative phosphorylation pathway was most significantly suppressed in the shRNA*Cs*-1429 cells,, in which a total of 10 differentially expressed proteins were enriched and were all downregulated by the abnormal expression of *Cs.* The downregulations of Ndufb5, Ndufv1 and Uqcrb were confirmed by immunoblotting. Meanwhile, the ATP levels of shRNA*Cs*-1429 cells were also reduced.

**Conclusions:**

These results suggest that low level expression of *Cs* induces the inhibition of oxidative phosphorylation pathway, which is responsible for the high level production of reactive oxygen species and low level of ATP, leading to the apoptosis of cochlear cells. This study may provide new theories for understanding and therapy of progressive hearing loss.

**Supplementary Information:**

The online version contains supplementary material available at 10.1186/s12953-022-00196-0.

## Background

HEI-OC1 (House Ear Institute-Organ of Corti 1) cells are derived from the auditory organ of transgenic mouse immortomouse™ and are immortalized at 33 °C/10% CO_2_ [1, 2]. They express specific molecular markers of cochlear hair cells, including prestin, Atoh1, myosin 7a, BDNF, *et. al*, and markers of supporting cells such as connexin 26 and fibroblast growth factor receptor. They have been widely used as an in vitro system to study cellular and molecular mechanisms related to hearing lose, such as cell death, apoptosis, survival, proliferation, senescence and autophagy, oxidative stress, etc. [3].

Citrate Synthase (CS) (EC 2.3.3.1) catalyzes the first step reaction of tricarboxylic acid cycle and plays an important role in substance metabolism and energy conversion. *Cs* gene mutation (locus *ahL4*) has been found to play an important role in progressive hearing loss of A/J mice [4]. The missense mutation of the third exon nucleotide of *Cs* gene (H55N) is the root of *ahl4*-related hearing impairment. It has been reported that the level of reactive oxygen species (ROS) was increased significantly accompanied by the upregulation of endoplasmic reticulum stress (ERS) markers in HEI-OCI cells with low-expressed *Cs* [5]. It provided a new probable theory for the mechanism of progressive hearing loss in A/J mice with *Cs* mutation. But it needs further studies to clarify details of the mechanism of ROS production, ERS and apoptosis mediated by the abnormal expression of *Cs*. This study further analyzed the differentially expressed proteins (DEPs) caused by low expression of *Cs* from the perspective of proteomics by iTRAQ proteomic quantitative techniques.

The technology of relative and absolute quantitative isobaric labelling (iTRAQ) combined with reverse phase liquid chromatography-tandem mass spectrometry (LC-MS/MS) is a powerful tool for proteomic studies and for maximizing the collection of complete protein information, which is characterized by high sensitivity, high resolution, and good repeatability [6]. iTRAQ combined with LC-MS/MS technology was used to screen differentially expressed proteins in HEI-OC1 cells with low-expressed *Cs* and normal-control HEI-OC1 cells, and Gene Ontology (GO) functional annotation enrichment analysis was carried out for differential proteins based on OMICSBean omics data consolidation and analysis cloud platform, and also KEGG pathway and protein-protein interaction (PPI) analysis.

## Methods

### Cells and the main reagents

HEI-OC1 (House Ear Institute-Organ of Corti 1) Cells were donated by F. Kalinec of House College of Ear Sciences in the United States and kept in the Key Laboratory of Genetic Hearing Disorders of Shandong Province. shRNA*Cs*-1429 cells with pGLV-H1-GFP + Puro-*Cs*1429 which contain shRNA sequences (5′-GCATGACGGAGATGAACTACT-3′) targeting mouse *Cs* gene were the low-expression *Cs* cell model constructed from HEI-OC1 cells in our laboratory, shRNA-NC cells with pGLV-H1-GFP + Puro-NC as negative control [5].

Dulbecco’s Modified Eagle’s Medium (DMEM) and fetal bovine serum (FBS) were purchased from Gibco USA. iTRAQ Reagent form AB Sciex LLC (USA). Other reagents are from Sangon Biotech (Shanghai, China) Co., Ltd.

### Cell culture and total protein extraction

Cells were cultured in high-glucose DMEM (FBS, 10%) medium at 33 °C and 10% CO_2_. The medium was changed every 2–3 days. Logarithmic growth phase cells were collected for lysis on ice with RIPA (PMSF, 1 mM) buffer. The collected cells were broken by ultrasound under low temperature with power of 80 W, ultrasound 1.0 s, and turned off 1.0 s, for a total of 3 min. After centrifugation at 12000 r·min^− 1^ and 4 °C for 20 min, the supernatants were collected as the total protein extract. The total protein concentration was determined by BCA protein assay (PC0020, Solarbio).

### Proteolysis and iTRAQ labeling

Total protein extraction (100 μg) was reacted with 120 μL reducing buffer (10 mM DTT, 8 M Urea, 100 mM TEAB, pH 8.0) on 10 K ultrafiltration tube at 60 °C for 1 h, and then reacted with the alkylation reagent iodoacetamide (IAA, 50 mM) in the dark for 40 min at room temperature. The filter was washed with 100 μL TEAB (100 mM) twice. 2 μl sequencing-grade trypsin (1 μg/μL) was used to digest proteins on filter of each tube at 37 °C for 12 h. The hydrolysis products were labeled with iTRAQ reagent (ABSCIEX) at room temperature for 2 h. The reaction was terminated by 200 μL of HPLC water for each sample. The labeling peptides solutions were lyophilized and stored at − 80 °C.

### Analysis and identification of reverse phase chromatography-mass spectrometry

The labeled polypeptide mixture was separated by reversed-phase chromatography. The mobile phase A was H_2_O-FA(99.9:0.1, v/v) and the mobile phase B was ACN-H_2_O-FA (80:19.9:0.1, v/v/v). The obtained samples were sampled on a C18 precolumn (PepMap C18, 100 μm × 2 cm, 5 μm) at a flow rate of 300 nL/min. The peptide segments were then gradient eluted with an analytical column (PepMap C18,, 75 μm × 50 cm, 2 μm) and detected by mass spectrometry. Cellular protein extractions and mass spectrometry analysis were performed in triplicate.

### Protein identification and quantification

The data were analyzed with The software Proteome DiscovererTM 2.2 (Thermo Company of the United States) on the basis of uniprot-Proteome_UP000000589-*mus Musculus* (Mouse) (Strain C57BL6J) database. The false-positive rate of peptides identification was controled under 1%. The parameter for searching library was set to: 1) Sample type: iTRAQ 8 plex (Peptide Labeled); 2) Cysteine alkylation: iodoacetamide; 3) Digestion: trypsin; 4) Trusted proteins: screening according to Score Sequest HT > 0 and unique peptide ≥1, and the blank value was removed.

### Bioinformatics analysis

Based on the screened trusted proteins, the FC value and *p*-value of difference significance of the comparison group were calculated. FC > 1.1 or FC < 10/11 and *p*-value < 0.05 were used as the standard to screen the trusted proteins with significant difference. Functional classification and GO enrichment analysis and KEGG pathway analysis were constituted by integration analysis cloud platform based on OmicsBean omics data. GO functional annotation includes three levels of analysis(http://www.geneontology.org): Biological Process, Cellular Component and Molecular Function. Target pathway analyses were identified with KEGG (https://www.genome.jp/kegg/). The functional PPI network was explored in STRING (http://string-db.org/) and pictured by Cytoscape (v3.7.1) software.

### Immunoblotting

The processes of the extraction of total proteins from cells and the concentration determination of total proteins were conducted as above. Equal amounts of proteins in each sample were loaded onto 10% sodium dodecyl sulfate polyacrylamide (SDS-PAGE) gel following with electrophoresis. Proteins were then transferred to polyvinylidene fluoride (PVDF) membrane (microporous). The membranes were incubated at 4 °C overnight with antibodies against *Cs* (ab96600, Abcam), Ndufb5 (23855–1-AP, Proteintech), Ndufv1(11238–1-AP, Proteintech) and Uqcrb(10756–1-AP, Proteintech) or β-actin (ab8227, Abcam) at a dilution of 1: 1000. Then the membranes were washed three times with TBST, followed with incubation with horseradish peroxidase-conjugated goat anti-rabbit secondary antibody (ab97051 1:5000, Abcam) at room temperature for 2 h. The bands of specific proteins were visualized by ECL kit (NOVLAND, Shanghai) and photographed in the chemiluminescence instrument (Clinx Science Instruments Shanghai, China). The intensity of every protein band was quantified by Image J software with that of β-actin as control.

### Intracellular ATP analysis

ATP levels of cells were measured using the ATP Assay Kit (Beyotime Biotechnology, Shanghai, China). Logarithmic growth phase cells were seeded into 6-well plates. 200 μL of lysis buffer for every well was added to lyse the cells when cells grew to 80%. The supernatant was collected and mixed with 100 μL of ATP test working solution in a black 96-well plate. Luminescence was measured by luminometer (Infinite M200 Pro, Tecan, Switzerland). The standard curves were generated with ATP standard solution (concentrations: 0.01, 0.03, 0.1, 0.3, 1, 3 and 10 μM) for each experiment. The ATP concentration was determined according the standard curve and standardized by protein concentration (BCA Protein Assay Kit, Beyotime Biotechnology, Shanghai, China).

### Statistical analysis

Statistical analysis was carried out by GraphPad Prism 9 software (GraphPad Software, San Diego, CA, USA). All the bars in the graph represent the means ± SEM from at least three independent experiments. For determining the significance of differences between two groups, Student’s t-test was applied. *P*-value < 0.05 was considered as statistically significant difference.

## Results

### Identification of differentially expressed proteins (DEPs) in cells of shRNA-NC and shRNACs-1429

**Fig. 1:**
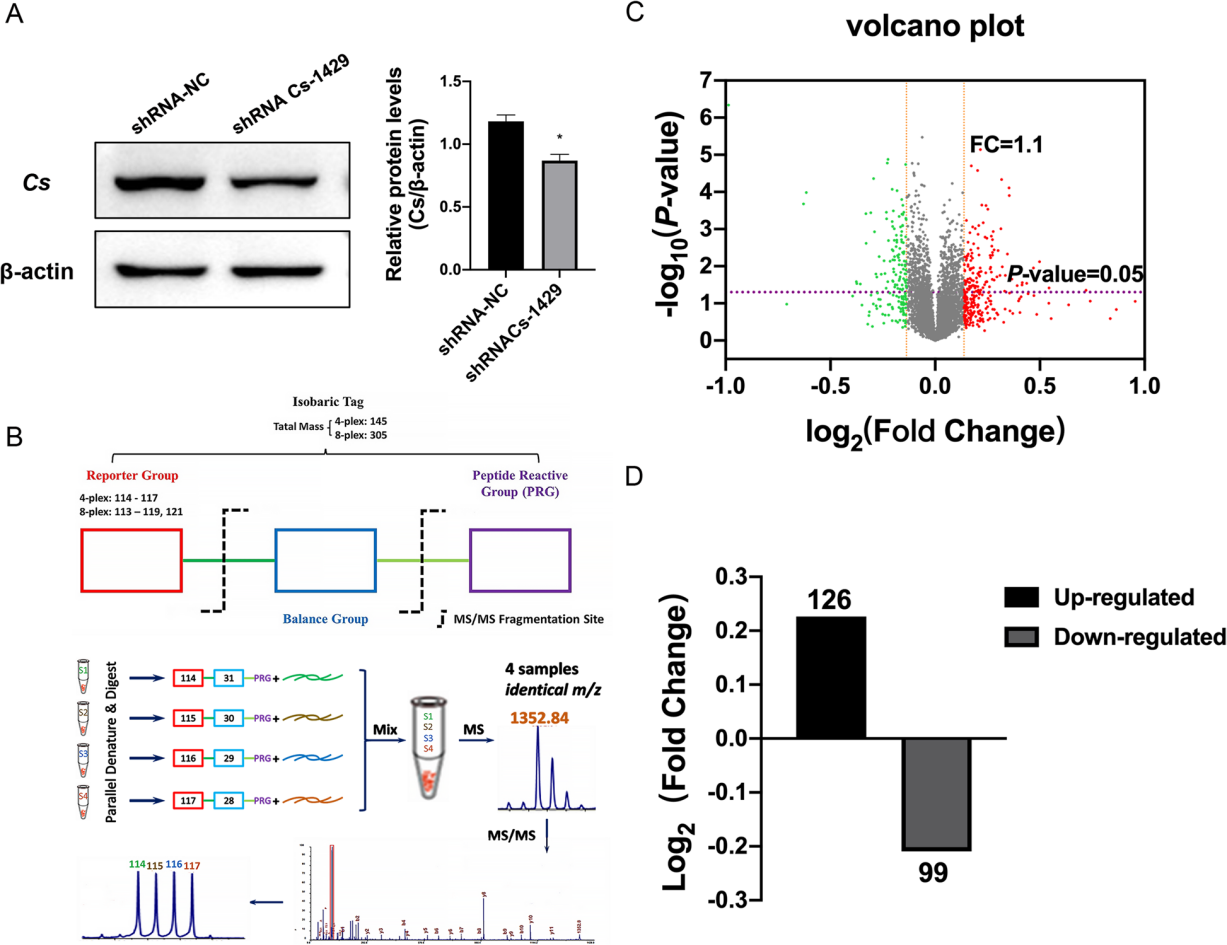
Identification of differentially expressed proteins between cells of shRNA-NC and shRNA*Cs*-1429. **A** The identification of the cell model shRNA*Cs*-1429 with low expression of *Cs*. The expression of *Cs* in HEI-OC1 cells was tested by Western blotting with β-actin as control. The expression level of *Cs* in shRNA*Cs*-1429 cells was remarkably decreased compared with that of shRNA- NC cells. **B** Schematic diagram of workflow for the iTRAQ-based approach. Total proteins were extracted from shRNA-NC cells and shRNA*Cs*-1429 cells, and part of the samples was taken for protein concentration determination and SDS-PAGE detection, and the other part was taken for trypsin enzymolysis and labeling, then the same amount of labeled samples were mixed for chromatographic separation, and finally the sample was analyzed by LC-MS/MS and data analysis. **C** The expression profiling changes of proteins from HEI-OC1 cells with of shRNA-NC or shRNA*Cs*-1429. Vocalno Plot indicated up-regulated and down-regulated proteins. Dots highlighted in red (up-regulated) or green (down-regulated) indicated proteins differentially expressed based on the criteria of ratio > 1.1 (or < 1/1.1) and *P*-value < 0.05. **D** The summary of differentially expressed proteins compared with shRNA-NC cells and shRNA*Cs*-1429 cells

To explore the alterations initiated by low expressed *Cs* in HEI-OC1 cells at the molecular levels, a proteomics analysis was performed on shRNA-NC and shRNA*Cs*-1429 cells. In total, 4574 proteins were identified in the present study. Based on the criteria of fold change > 1.1 (or < 1/1.1) and *P*-value < 0.05, 225 proteins were identified with differential expression between shRNA-NC and shRNA*Cs*-1429 cells, among which 126 proteins were significantly up-regulated and 99 proteins significantly down-regulated. The details of differentially expressed proteins compared with shRNA-NC cells and shRNA*Cs*-1429 cells (Supplementary Table 1). Volcano Plot analysis was conducted to visualize the DEPs between shRNA-NC and shRNA*Cs*-1429 cells. (Fig. 1).

### Gene ontology (GO) functional analysis and enrichment of DEPs

**Fig. 2:**
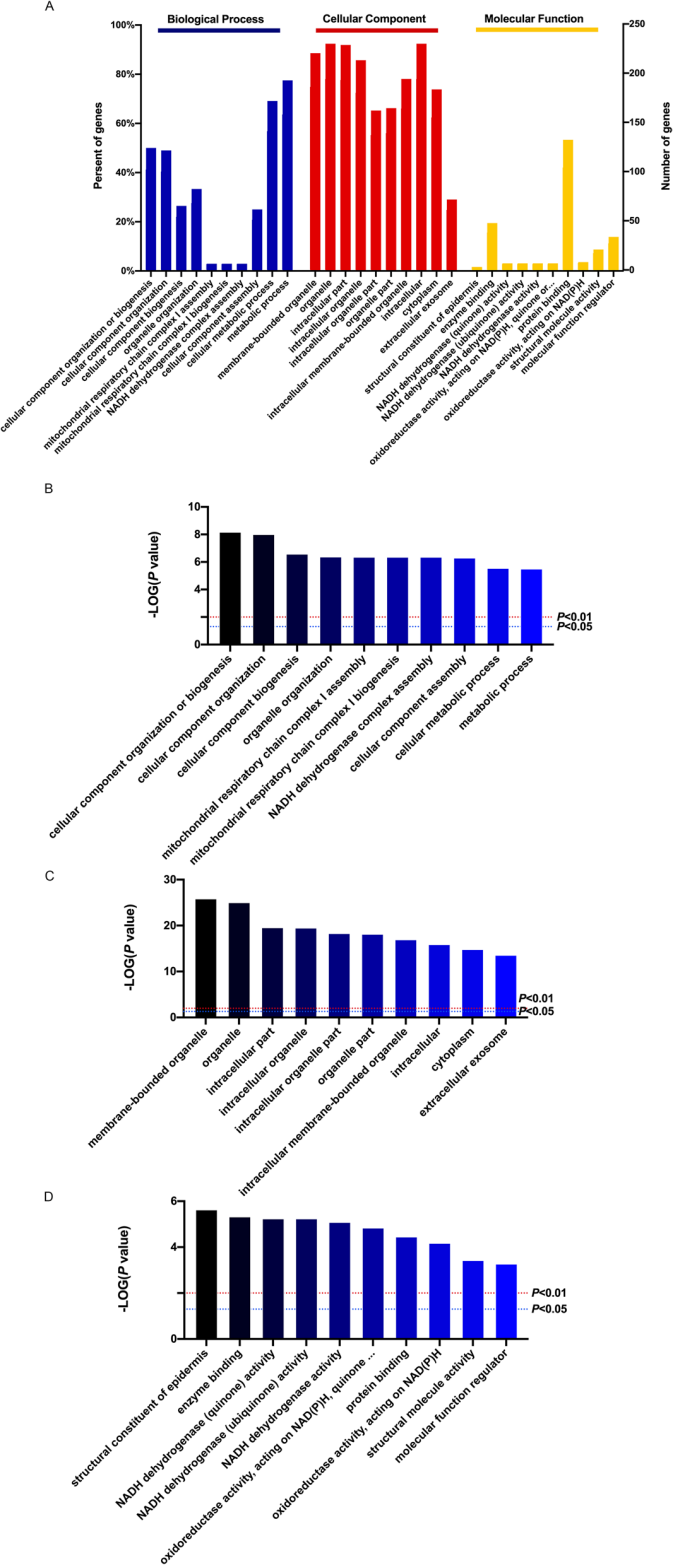
GO functional annotation and enrichment analysis of differentially expressed proteins. **A** The summary of GO functional annotation. The abscissa of items of each category is in order from left to right according to the *P*-value, the more significant the left is, and the ordinate represents the number of differential proteins contained in each item and the percentage of the total differential proteins were performed on the screened differential proteins. The top 10 items of BP, CC and MF enrichment analysis significance were represented repsectively by (**B**), (**C**) and (**D**)

In order to further analyze the functional characteristics of the differential expressed proteins, the Gene Ontology functional annotation and enrichment analysis were performed on the screened differential expressed proteins based on the Integrate analytics cloud platforms of the OmicsBean omics data. Functional annotation of GO includes three levels of analysis: Biological Process (BP), Cellular Component (CC) and Molecular Function (MF). The items with significant enrichment in the three classifications were screened (*P* < 0.0001). 34 biological process items (Supplementary Table 2), 8 molecular function items (Supplementary Table 3), 61 cellular component items (Supplementary Table 4) were found. The diagram of GO enrichment analysis shows the top 10 items of BP, CC and MF in the significance of enrichment analysis. (Fig. 2).

### KEGG pathway analysis and enrichment DEPs

**Fig. 3:**
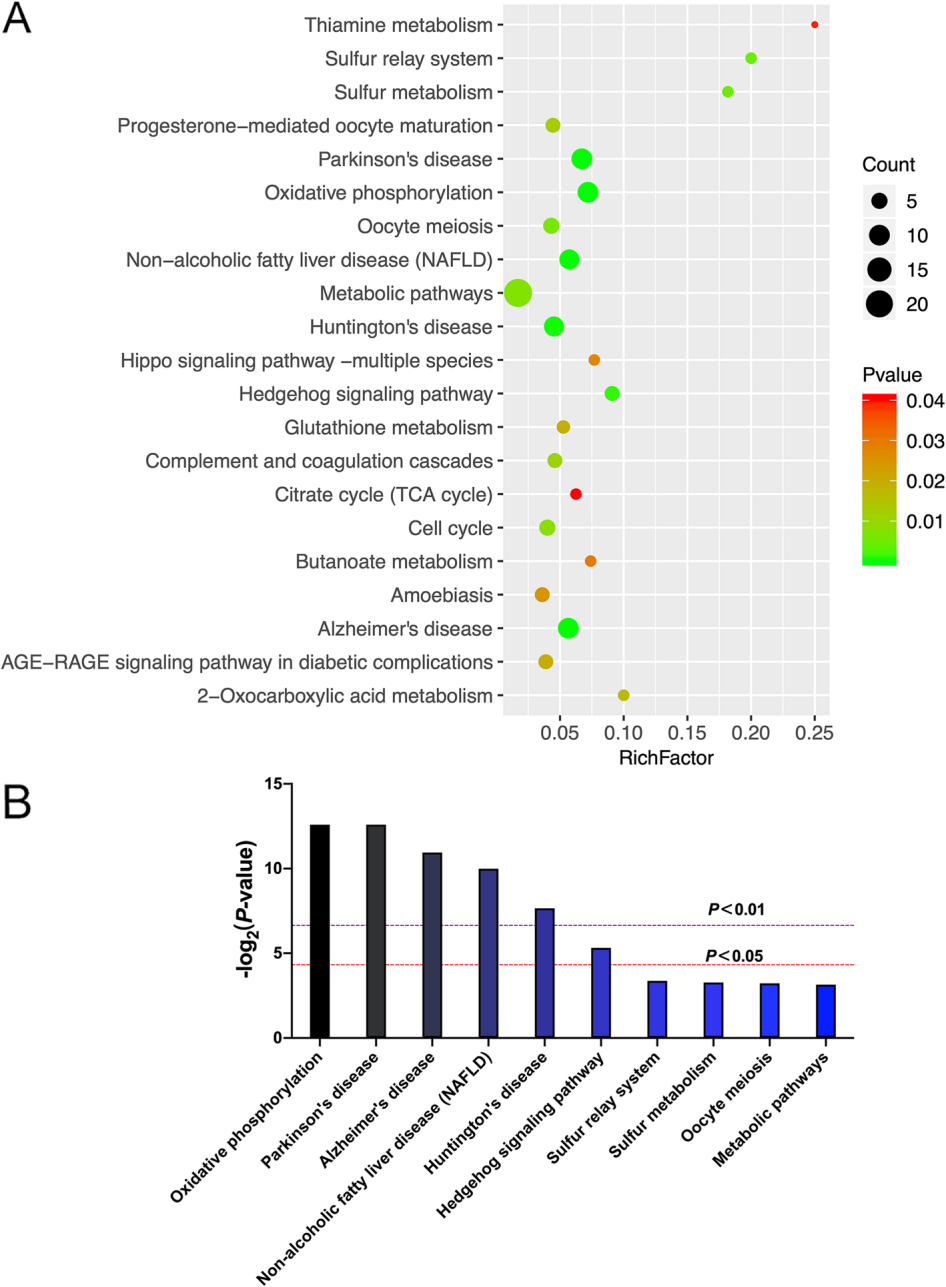
KEGG pathway analysis and enrichment results. **A** Bubble plots of KEGG (*P* < 0.05) enrichment. In the X-axis of the figure, the Rich Factor is the enrichment factor (the number of differential genes enriched in a pathway/the total number of genes annotated in the pathway). The items with larger bubbles contain more differential genes, and the color of bubbles represents the *P* value. The smaller the enrichment *P* value is, the greater the significance is. **B** The top ten KEGG pathways with significant differences are shown. The *P* value indicated by the purple line is 0.01, and the *P* value indicated by the red line is 0.05

KEGG is a database of pathway analyses that systematically analyze the metabolic pathways of proteins in cells and the functions of these proteins. The OMICSBean omics data were integrated and analyzed on the cloud platform, and KEGG pathway analysis (Supplementary Table 5) was conducted on the screened differential proteins. Top 10 KEGG pathways of significance were shown in Fig. 3.

Based on the Integrate analytics cloud platforms of the OmicsBean omics data, the KEGG pathway enrichment analysis showed that 116 differential proteins were involved in 21 biological signaling pathways (*P* < 0.05), and the differential proteins were mainly enriched in Oxidative phosphorylation, Parkinson’s disease, Alzheimer’s disease, Non-alcoholic fatty liver disease (NAFLD), Huntington’s disease, Hedgehog signaling pathway, et al. The largest number of DEPs was enriched in metabolic pathway, including 22 proteins. Oxidative phosphorylation (OXPHOS) pathway was the most significant signaling pathway.

### PPI analysis of DEPs and HUB gene screening

**Fig. 4:**
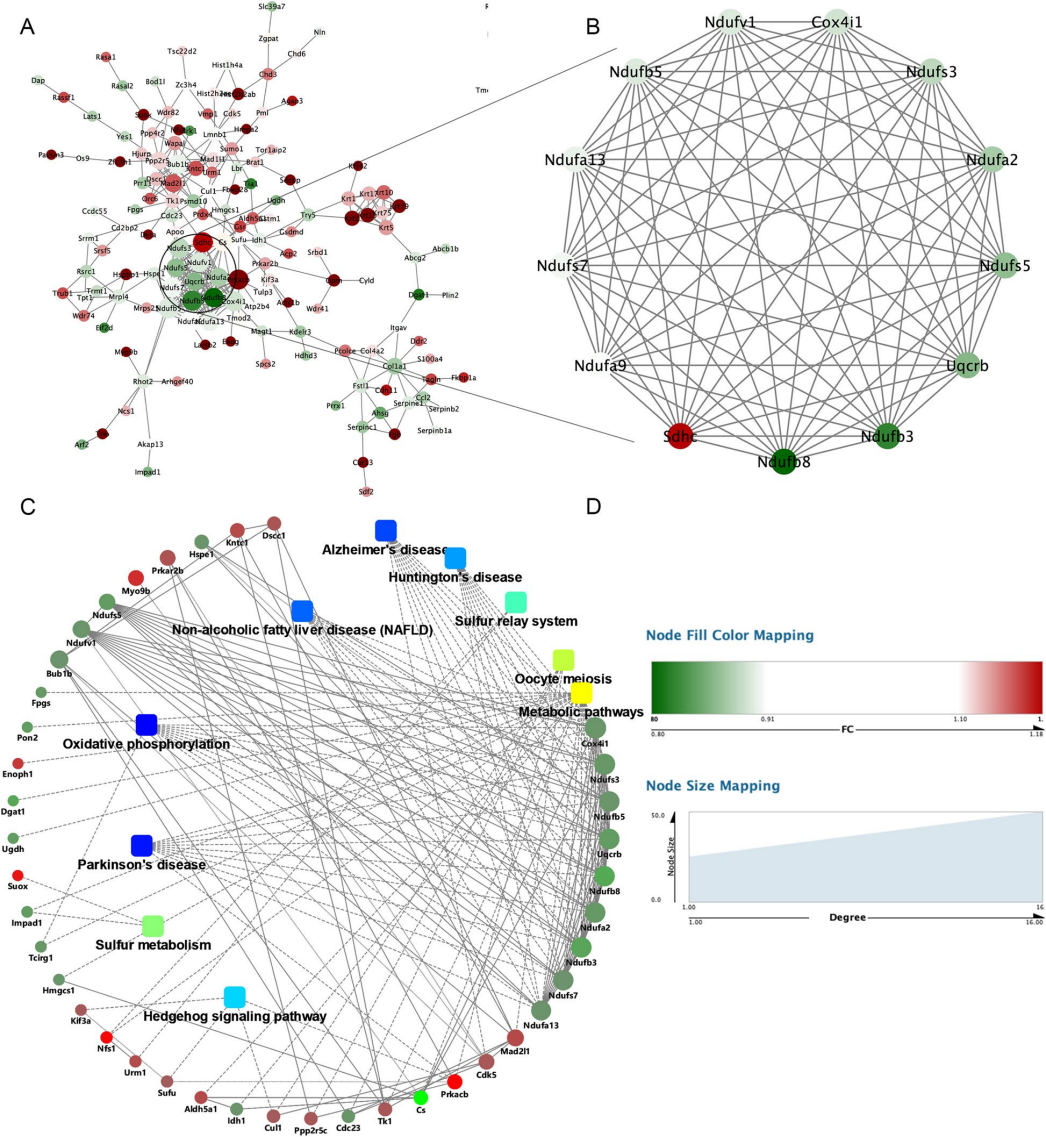
The proteins interactions between differential proteins and KEGG pathways. **A** The protein-protein interaction (PPI) network obtained from the STING database containing 43 nodes and 150 edges. **B** The PPI of the largest cluster containing 13 nodes and 88 edges. **C** The top ten significant KEGG enrichment pathways and the interaction network of the proteins interacting with them. **D** The legends of the pictures upon. The dots represent genes, red represents up-regulated expression, and green represents down-regulated expression. Rounded rectangles of (**C**) represent signaling pathways. Blue represents higher significance and yellow represents lower significance. The straight lines represent interactions, the solid lines represent protein-protein interactions, and the dashed lines represent metabolic pathways -- protein-protein interactions

PPI analysis of 225 DEPs was performed with the STRING database, and the protein network was visualized by Cytoscape software. Cytohubba plug-in screened out the first 10 HUB proteins according to the number of nodes, including Ndufb3, Ndufa9, Ndufs7, Ndufs3, Ndufb8, Ndufv1, Ndufb5, Ndufs5, Ndufa13, Ndufa2, all of which were decreased in shRNA*Cs*-1429 cells. These proteins are the subunits of complex I in OXPHOS. The analysis of protein interactions between DEPs and KEGG pathways was performed based on the Integrate analytics cloud platforms of the OmicsBean omics data. The interaction network between DEPs and the top ten KEGG pathways was showed in Fig. 4 C and supplementary Table 6.

### Oxidative phosphorylation pathway was suppressed in shRNACs-1429

**Fig. 5:**
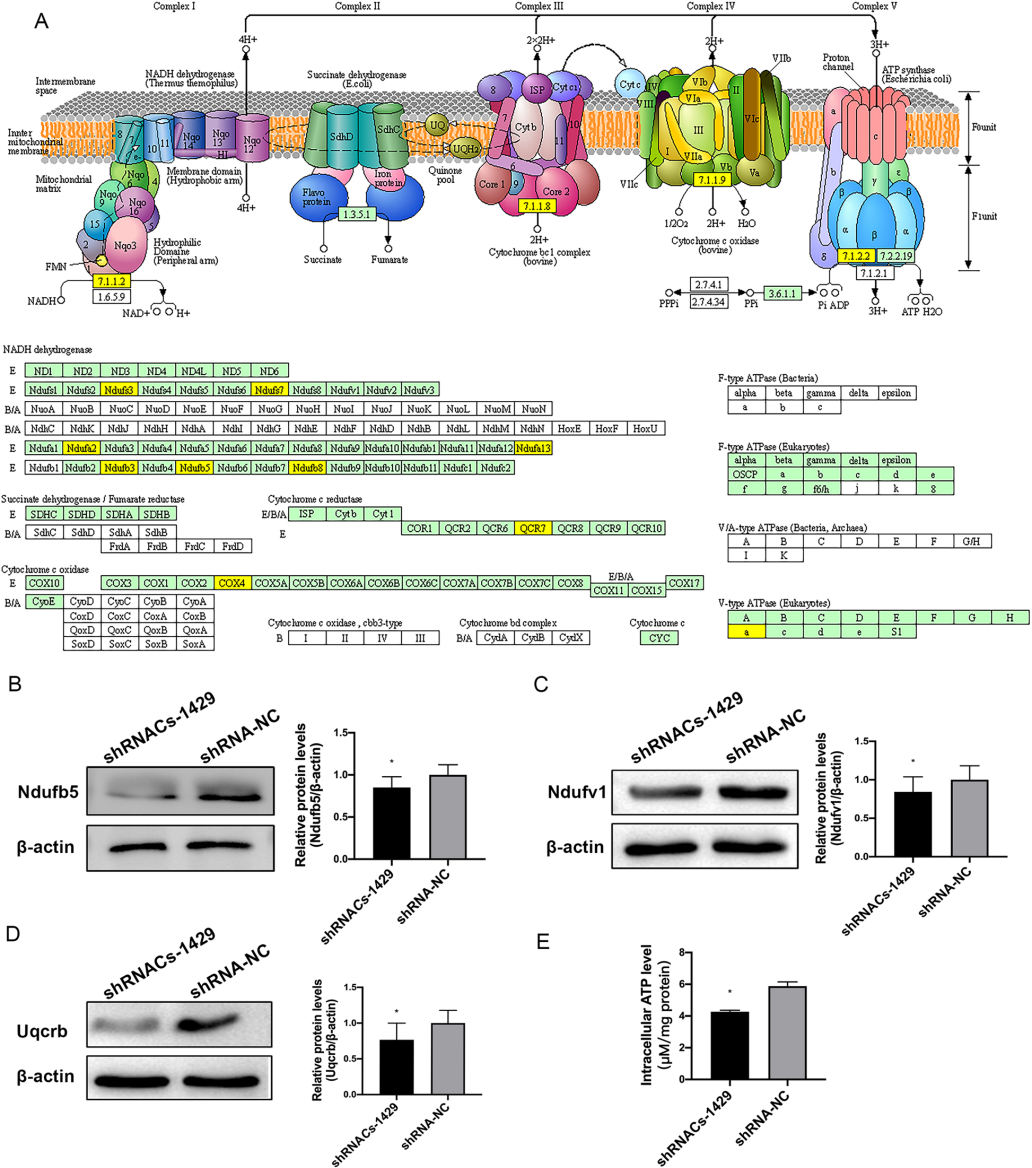
Oxidative phosphorylation pathway was suppressed in shRNA*Cs*-1429. **A** Oxidative phosphorylation pathway. Blue shows the differentially expressed proteins in OXPHOS pathway, and all were down-regulated in shRNA*Cs*-1429 cells. The decreased expression of Ndufb5 (**B**), Ndufv1 (C) and Uqcrb (**D**) was detected by western blotting in shRNA-1429 cells compared with shRNA-NC, with β-actin as reference. Data presented as mean ± SE of triplicate experiments. *P < 0.05, **P < 0.01. **E** The levels of intracellular ATP in shRNA*Cs*-1429 cells were significantly decreased compared to those in shRNA-NC cells (*P < 0.05)

Based on the above bioinformatics analysis results, we focused on the oxidative phosphorylation signaling pathway, because 10 differentially expressed proteins were enriched in the oxidative phosphorylation signaling pathway (Fig. 5), which are important components of mitochondrial oxidative respiratory chain complex I, III, IV and V. It is worth of mentioning that the expressions of 12 proteins were all differentially down-regulated in shRNA*Cs*1429 cells detected by iTRAQ experiment, which indicated that the OXPHOS pathway was suppressed in the HEC-OC1 cells with low-expressed *Cs*. In order to confirm the data from iTRAQ proteomics analysis, the expression level of 3 proteins, including Ndufb5, Ndufv1 and Uqcrb, were detected by immunoblotting (Fig. 5). The results showed that the expression levels of these three proteins were significantly down-regulated in shRNA*Cs*-1429 cells compared with shRNA-NC cells, which were consistent with those determined using the iTRAQ labeled LC-MS/MS system. Furtherly, the levels of intracellular ATP were examined, and the results showed that the production of ATP in shRNA*Cs*-1429 cells was significantly reduced compared with shRNA-NC cells, which may be caused by the inhibition of OXPHOS pathway.

## Disscusion

CS is the key enzyme of the tricarboxylic acid cycle, catalyzing the condensation of acetyl-Coenzyme A and oxaloacetate to citric acid, controlling the entrance of the tricarboxylic acid cycle and playing a decisive role in regulating energy generation. Several lines of evidence had been provided that a missense mutation of *Cs* is responsible for the hearing loss of A/J strain mice [4]. A/J strain mice have been widely used in the study of the anatomical, physiological, pathological and molecular mechanisms of age-related hearing loss (AHL) [7]. The onset of hearing loss is much earlier, showing an elevated hearing threshold as early as 25 days of age [8]. Genetic studies of hearing loss in A/J mice revealed an additional AHL locus, named *ahl* 4, on the distal-most 5.5 Mb chromosome (Chr) 10 [4, 7]. The *rs29358506* SNP in the third exon 3 of *Cs* gene (11 exons, cDNA 1395 nt, encoding 464 amino acids) is the root cause of *ahl* 4-related hearing impairment which resulted in a missense mutation (H55N) of *Cs*. Our previous studies have shown that the mRNA levels of apoptosis-related genes (caspase-3 and caspase-9, etc.) were increased in inner ears of A/J mice at postnatal day 1, and anti-apoptosis treatment can reduce the hearing threshold of A/J mice to some extent. Furthermore, downregulation of *Cs* expression HEI-OC1 cells significantly increased the expression of Caspase-3, indicating that the low expression of *Cs* could cause the enhancement of cell apoptosis signal. Similarly, a decrease in the activity of CS was detected in patients with Alzheimer’s disease (AD) [9]. Mitochondrial dysfunction caused by low activity CS are related to AD [10].

In the present study, 225 proteins were confirmed as DEPs between shRNA-NC and shRNA*Cs*-1429 cells, including 99 proteins downregulated and 126 proteins upregulated. The following bioinformatics analysis showed that the first ten HUB proteins were enriched in the oxidative phosphorylation signaling pathway and all downregulated differentially. This suggested that low expression of *Cs* lead to the dysregulation of oxidative phosphorylation which is one of the important causes of mitochondrial dysfunction. Mitochondrial dysfunction is a typical feature of aging diseases and aging-related degenerative diseases, such as Alzheimer’s disease and Parkinson’s disease [11]. In our study, the top 3 KEGG pathways were shown as DEPs were oxidative phosphorylation, Parkinson’s disease and Alzheimer’s disease. This indicated mitochondrial dysfunction occurred in shRNA*Cs*-1429 cells. Ongoing mitochondrial dysfunction resulted in cell dysfunction and death [12], which was reported in our previous work that the proliferation ability of shRNA*Cs*-1429 cells was decreased and apoptosis was increased [5]. Mitochondrial dysfunction also was a important cause of hearing loss because of the disorder or death of cochlear cells [13].

The mitochondrial oxidative phosphorylation system (OXPHOS) generates ATP by phosphorylating ADP, which occurs in conjunction with the transit of electrons from reducing equivalents (i.e., NADH, FADH_2_) down the electron transport chain (ETC), a series of transmembrane protein complexes in the mitochondrial inner membrane [14]. The electron transport chain is composed with four enzyme multi-subunit complexes, complex I (NADH-Coenzyme Q reductase), complex II (succinate-Coenzyme Q reductase), complex III (ubiquinol-cytochrome c reductase or cytochrome bc1 complex) and complex IV (cytochrome c oxidase) [15]. Complex I is the largest component of respiratory chain, transporting two electrons from NADH to reduce ubiquinone. In our study, 9 subunits of complex I were detected as DEFs with downregulated expression in shRNA*Cs*-1429 cells. Ndufv1 is one of the core subunits of the dehydrogenase domain of complex I, catalyzing the NADH oxidation by a non-covalently bound flavin-mononucleotide (FMN) [16]. NDUFS7 involves in the formation of a binding pocket in which electrons are transferred along iron-sulfur (FeS) to ubiquinone. Ndufs3, Ndufb5, Ndufa13, Ndufa2, Ndufb8, Ndufb3 and Ndufv5, as accessory subunits of complex I, involve in respiratory chain assembly. The downregulation of these proteins was detected in our study, which indicated the depress of electrons transfer in the mitochondrial respiratory chain of shRNA*Cs*-1429 cells. Complex III (Cytochrome b-c1 complex) transfers electrons from QH2 to cytochrome c coupling with the translocation of protons，and is composed of three highly conserved core subunits and eight supernumerary subunits [17]. Uqcrb, also named as Cytochrome b-c1 complex subunit 7, was found to be significantly down-regulated in shRNA*Cs*-1429 cells. Uqcrb is one of supernumerary subunits, and required for assembly, stability, modulation and regulation of complex III [18]. Complex IV (Cytochrome c oxidase) transfers electrons from cytochrome c to oxygen, catalyzing the production of H_2_O. Cytochrome c oxidase subunit 4 is the largest of the accessory subunits. It is responsible for the allosteric inhibition of complex IV by binding ATP with increased ATP/ADP ratios [19]. Cytochrome c oxidase subunit 4 isoform 1(Cox4i1) was decreased in shRNA*Cs*-1429 cells. The generation of ATP was decreased in shRNA*Cs*-1429 cells which may be caused by the downregulation of multiple subunits of OXPHOS.

During the process of electrons transfer to O_2_ along ETC, some of the electrons leaking out noncovalently reduces O_2_ to hydroxyl radicals [2] and superoxide radicals, which can be immediately dismutated into hydrogen peroxide by superoxide dismutase [20]. The generation of mitochondrial ROS is closely related with complex I and complex III [21], especially complex I [22]. The production of ROS was increased when complex I was inhibited by its specific inhibitor Rotenone [23]. In our study, 9 subunits were decreased in low-expressed *Cs* cells, which resulted in the inhibition of complex I. Additionally, ROS was detected increasingly in our previous study [5]. It indicated that the inhibition of complex I induced by the low expression of *Cs* in HEI-OC1 cells may be responsible for the enhancement of ROS production. Increase of ROS production results oxidative stress, leading to mitochondria dysfunction and cellular apoptosis in patients with calcific aortic valve disease, atherosclerosis, type-II diabetes and chronic kidney disease [24]. Efficient preventing reactive oxygen species (ROS) formation had therapeutic implications in ischemia-reperfusion (IR) injury [25].

## Conclusions

It was reported previously that the elevated level of reactive oxygen species was closely related to the low expression of *Cs* not only in 293 T cells [26] but also in HEI-OC1 cells [5]. ROS, as is well known, is mainly produced from OXPHOS, especially complex I and complex III in mitochondria. The disruption of OXPHOS can lead to the enhancement of ROS. The downregulation of 10 subunits of OXPHOS was detected by ITRAQ- proteomic analysis in *Cs* low-expressed HEI-OC1 cells in this study. It was supposed that the excess ROS is mediated by the inhibition of OXPHOS, which is induced by the low expression of *Cs* in HEI-OC1 cells. The oxidative damage of HEI-OC1 cells caused by ROS led to apoptosis in vitro, which indicated that the loss of outer hair cells (OHC) was caused by the excess ROS induced by the abnormal expression of *Cs* in A/J mice. This assumption will be verified in our following studies to reveal the mechanism of progressive hearing loss in A/J mice in the hope of providing new theories for understanding and therapy of age-related hearing loss.

## Supplementary Information

**Additional file 1.** Supplementary material for qRT-PCR.

**Additional file 2: Supplementary Material 2.**

**Additional file 3: Supplementary Table 1.**

**Additional file 4: Supplementary Table 2.**

**Additional file 5: Supplementary Table 3.**

**Additional file 6: Supplementary Table 4.**

**Additional file 7: Supplementary Table 5.**

**Additional file 8: Supplementary Table6.**

## Data Availability

All data generated or analysed during this study are included in this published article and its supplementary information files.

## Abbreviations

Cs: Citrate Synthase
ROS: Reactive oxygen species
DEPs: differentially expressed proteins
PPI: Protein–protein interaction
ERS: Endoplasmic reticulum stress
GO: Gene Ontology
FBS: Fetal bovine serum
DMEM: Dulbecco’s Modified Eagle’s Medium
BP: Biological Process
CC: Cellular Component
MF: Molecular Function
OXPHOS: Oxidative phosphorylation
AHL: Age-related hearing loss
AD: Alzheimer’s disease
