# Supplementary material for: Effect of downregulated citrate synthase on oxidative phosphorylation signaling pathway in HEI-OC1 cells
Source: Proteome Sci. 2022 Sep 7;20:14. doi: 10.1186/s12953-022-00196-0 (PMC9450364; doi:10.1186/s12953-022-00196-0)
Supplement: Supplementary file 1 — Additional file 1. Supplementary material for qRT-PCR. [file 12953_2022_196_MOESM1_ESM.docx]

**The** **qRT-PCR results of Ndufb5, Ndufv1 and Uqcrb**

**Table 1. The primers for quantitative real-time PCR**

| **Gene** | **Forward Primer (5**′**-3**′**)** | **Reverse Primer (5**′**-3**′**)** |
| --- | --- | --- |
| *β*-actin | GTGGGAATGGGTCAGAAGGA | CTTCTCCATGTCGTCCCAGT |
| Ndufb5 | CAAGAGACTGTTTGTCGTCAAGC | TGTTCACCAGTGTTATGCCAAT |
| Ndufv1 | GACGGGGTGACTGGTACAAG | CGCATGATCTCTCGGTCCTT |
| Uqcrb | CGGCGTGTCCTCTAGGACTTA | CCACTTTCGAAAACCATCCA |


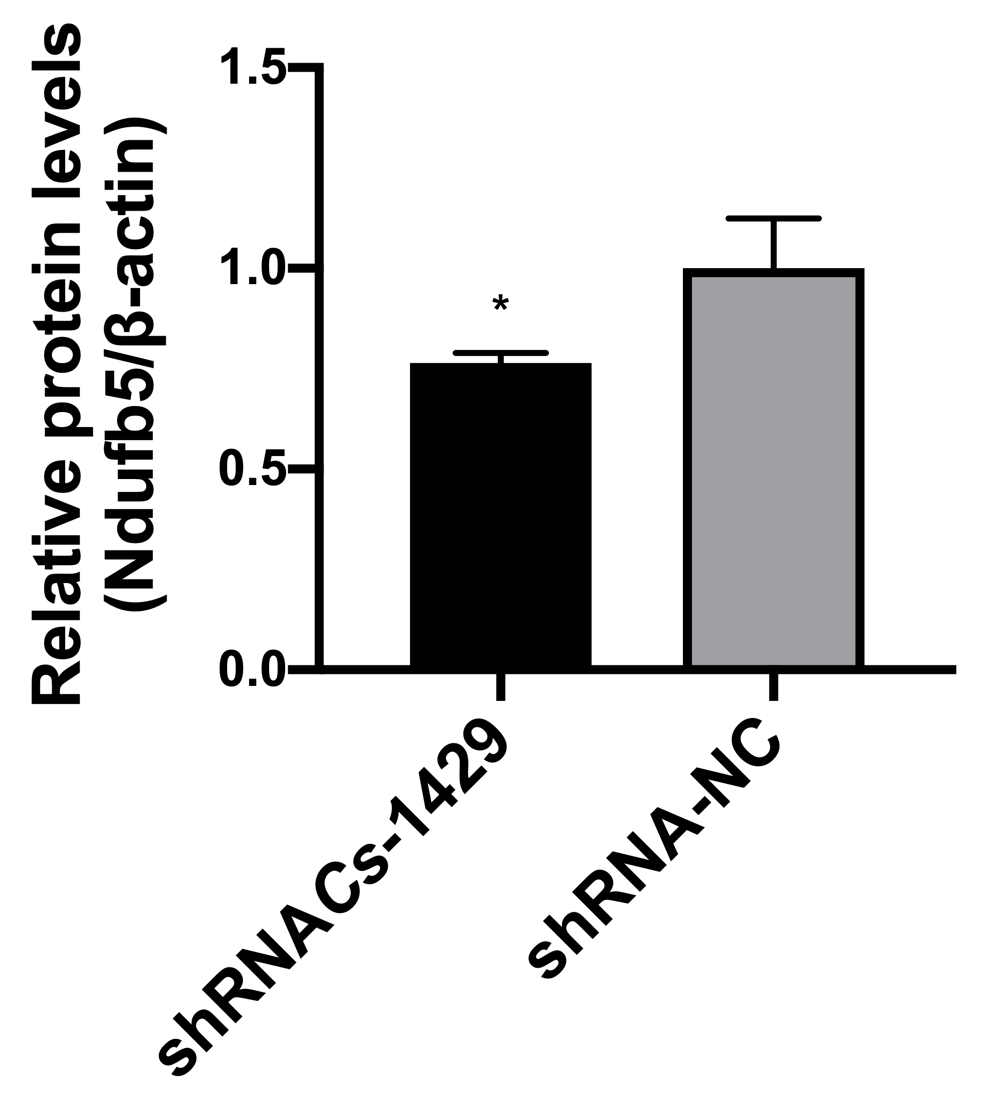

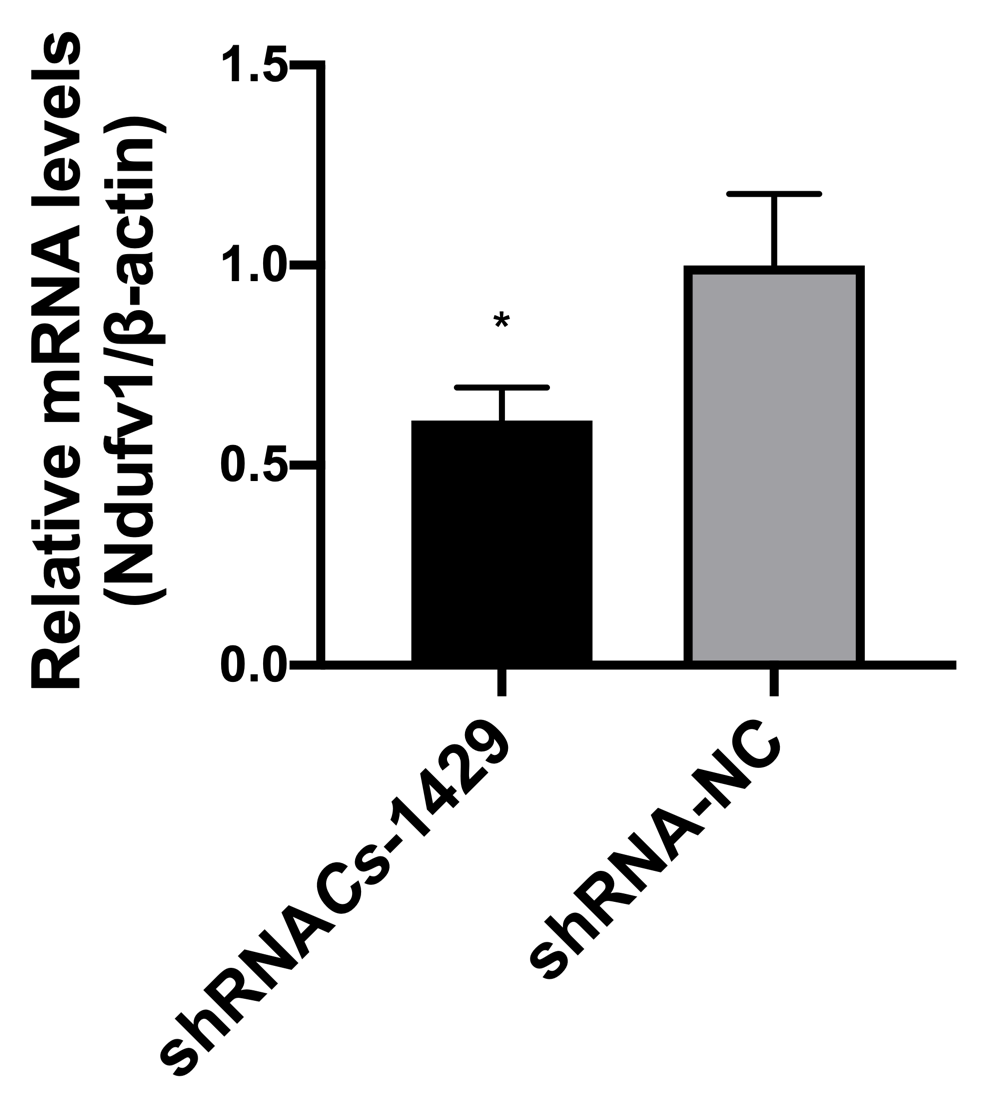

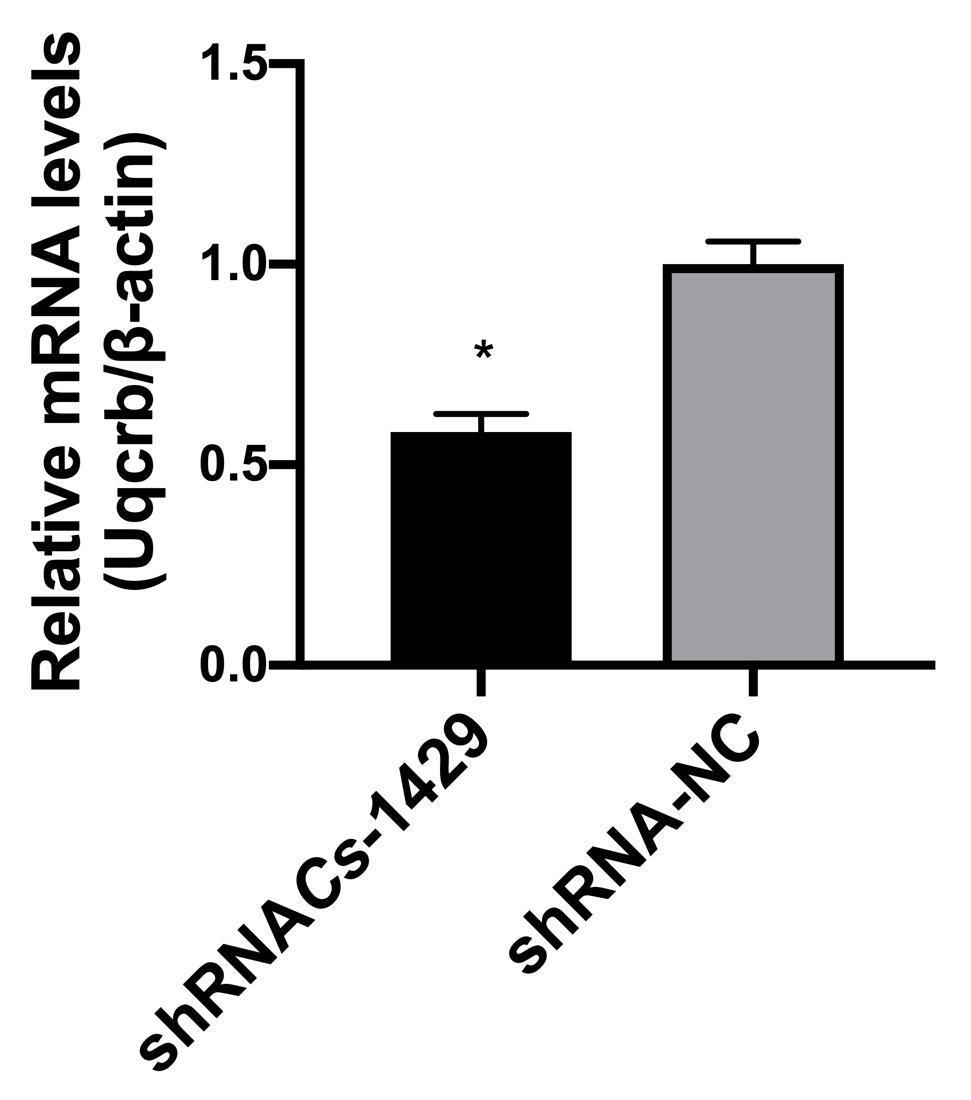


**Figure 1. Relative expression levels of selected three genes related to oxidative phosphorylation pathway by quantitative real-time PCR**
