# Supplementary figures and images for: Effect of downregulated citrate synthase on oxidative phosphorylation signaling pathway in HEI-OC1 cells
Source: Proteome Sci. 2022 Sep 7;20:14. doi: 10.1186/s12953-022-00196-0 (PMC9450364; doi:10.1186/s12953-022-00196-0)

## Supplementary material-2

Figure 1 (A)

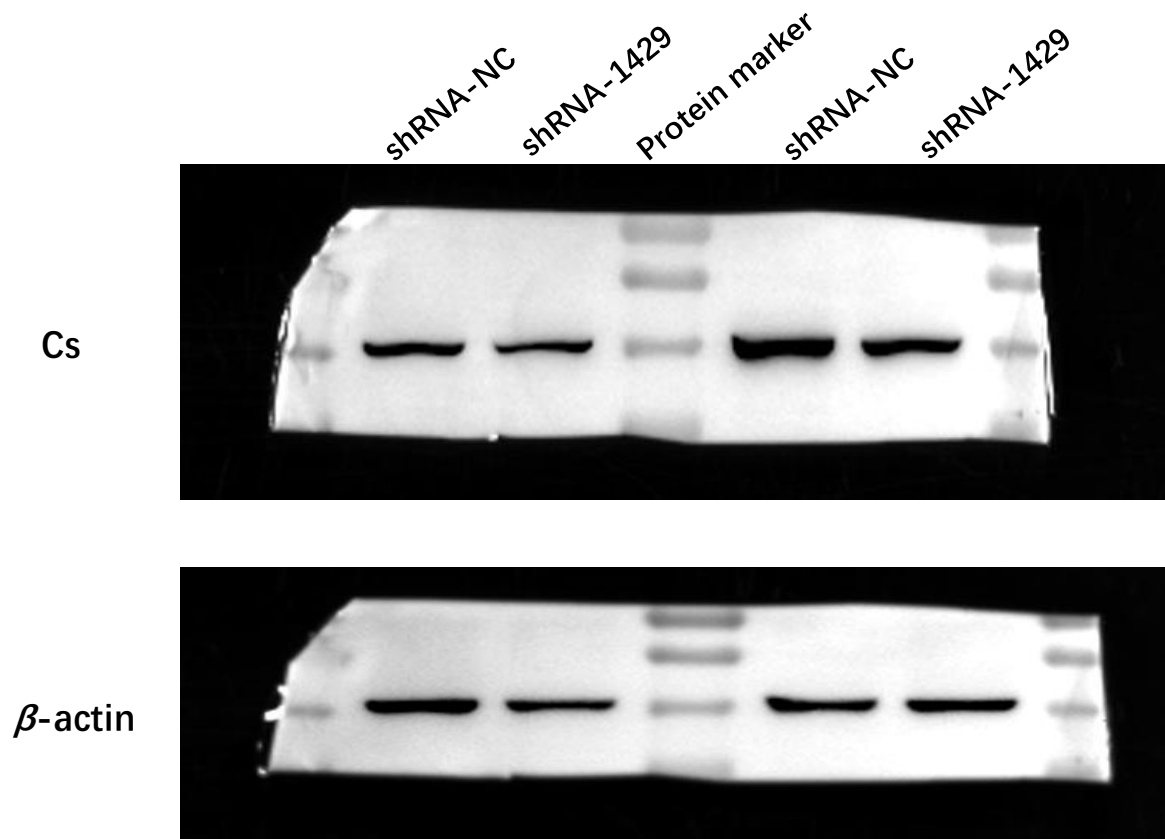

Figure 5 (B)

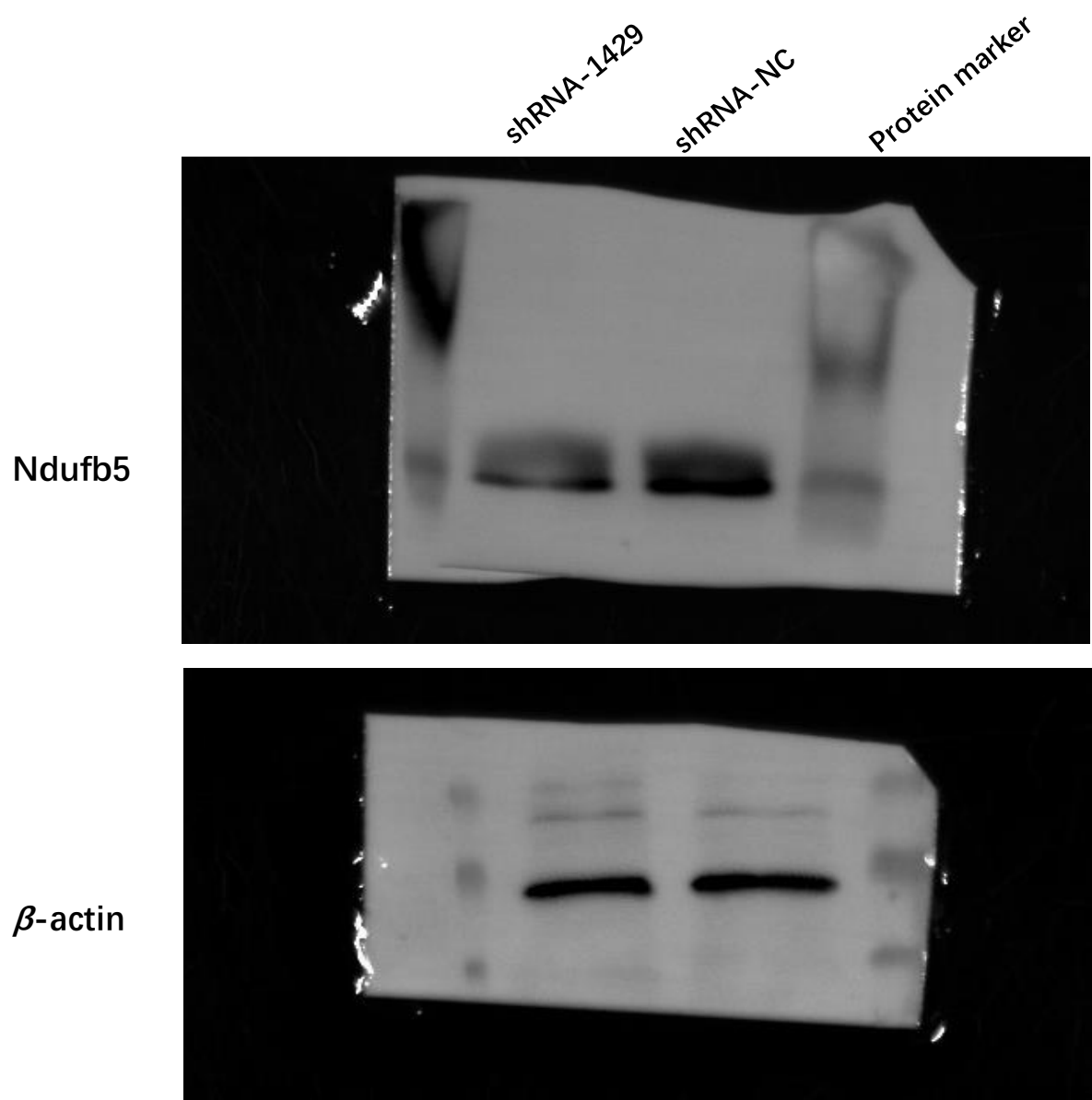

Figure 5 (C)

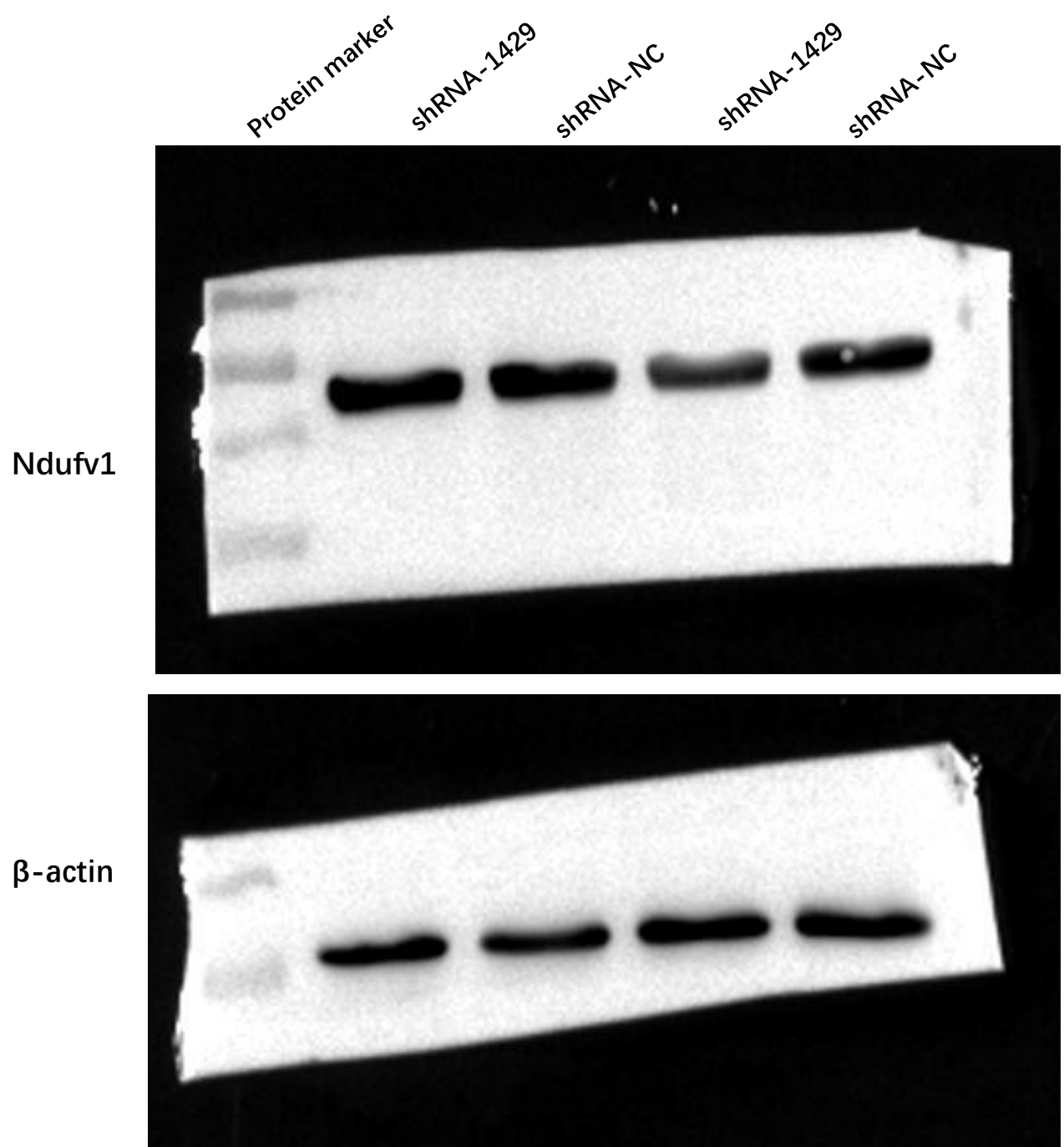

Figure 5 (D)

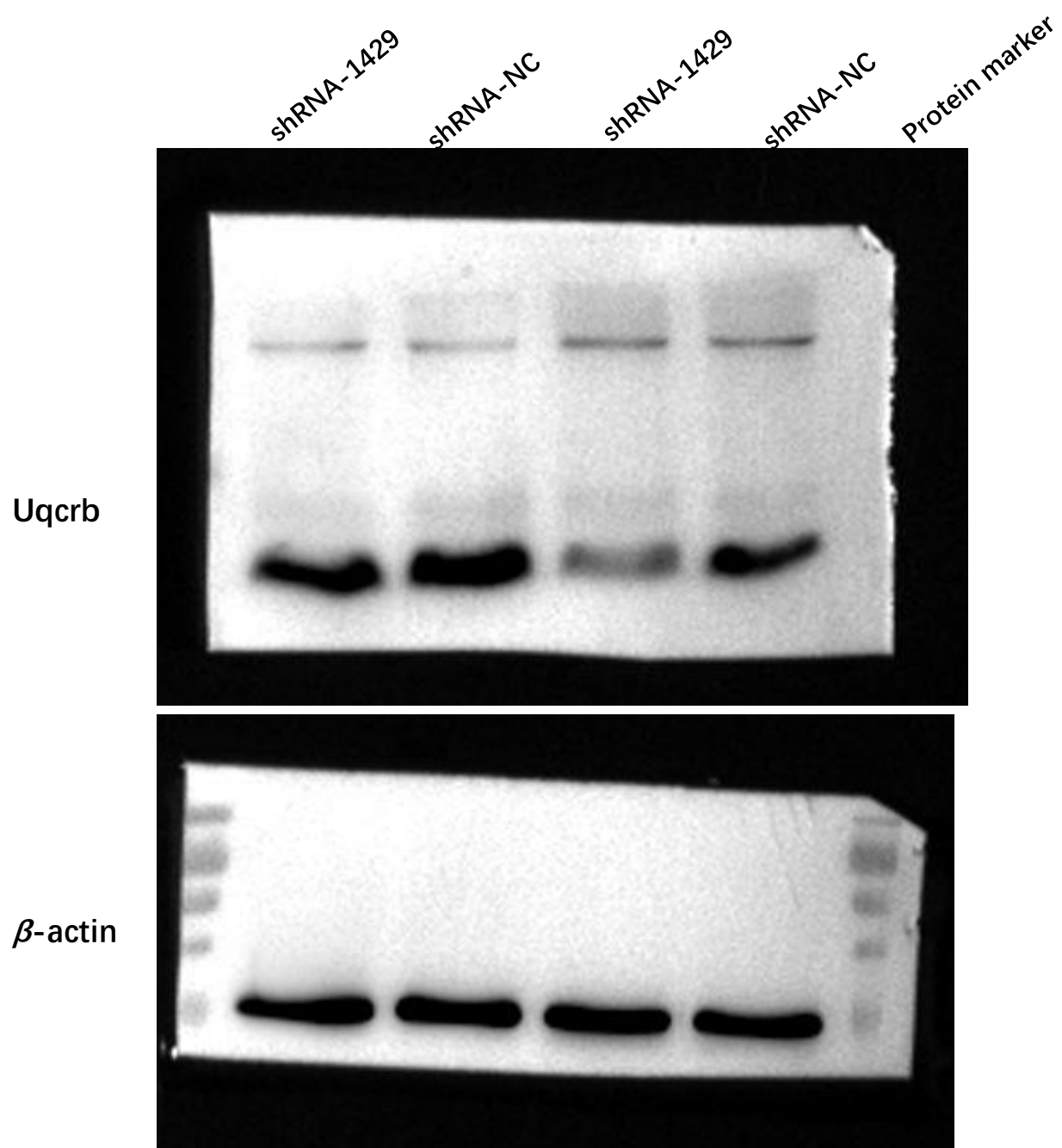

Supplement: Supplementary file 2 — Additional file 2: Supplementary Material 2. [file 12953_2022_196_MOESM2_ESM.pdf]
